# A scoping review of machine learning models to predict risk of falls in elders, without using sensor data

**DOI:** 10.1186/s41512-025-00190-y

**Published:** 2025-05-06

**Authors:** Angelo Capodici, Claudio Fanconi, Catherine Curtin, Alessandro Shapiro, Francesca Noci, Alberto Giannoni, Tina Hernandez-Boussard

**Affiliations:** 1 Department of Health Management (Direzione Sanitaria), IRCCS Istituto Ortopedico Rizzoli, Bologna, 40127 Italy; 2 Interdisciplinary Research Center for Health Science, Sant’Anna School of Advanced Studies, Pisa, 56127 Italy; 3 Department of Electrical Engineering and Information Technology, ETH Zurich, Zurich, Switzerland; 4 Department of Surgery, Veterans’ Affairs Palo Alto Healthcare System, Palo Alto, CA USA; 5 Institute of Life Sciences, Scuola Superiore Sant’Anna, Pisa, Italy; 6 Division of Cardiology and Cardiovascular Medicine, Fondazione Toscana Gabriele Monasterio, Via Moruzzi, 1, Pisa, 56124 Italy; 7 Department of Medicine, Stanford Center for Biomedical Informatics Research (BMIR), Stanford University, Stanford, USA

**Keywords:** Artificial intelligence, Machine learning, Elders, Falls, Sensorless, Scoping review

## Abstract

**Objectives:**

This scoping review assesses machine learning (ML) tools that predicted falls, relying on information in health records without using any sensor data. The aim was to assess the available evidence on innovative techniques to improve fall prevention management.

**Methods:**

Studies were included if they focused on predicting fall risk with machine learning in elderly populations and were written in English. There were 13 different extracted variables, including population characteristics (community dwelling, inpatients, age range, main pathology, ethnicity/race). Furthermore, the number of variables used in the final models, as well as their type, was extracted.

**Results:**

A total of 6331 studies were retrieved, and 19 articles met criteria for data extraction. Metric performances reported by authors were commonly high in terms of accuracy (e.g., greater than 0.70). The most represented features included cardiovascular status and mobility assessments. Common gaps identified included a lack of transparent reporting and insufficient fairness assessments.

**Conclusions:**

This review provides evidence that falls can be predicted using ML without using sensors if the amount of data and its quality is adequate. However, further studies are needed to validate these models in diverse groups and populations.

**Supplementary Information:**

The online version contains supplementary material available at 10.1186/s41512-025-00190-y.

## Background

Falls in elders are common and potentially devastating events [1]. Falls are a major cause of morbidity and mortality in elderly populations with 32,000 elders dying as a result each year [2]. In addition to the human cost caused by falls, the monetary costs are staggering, amounting to US $50 billion in 2018 alone [3, 4]. The high risk of falls is highlighted by in-hospital falls being included as a “never” event — occurrences that are clearly identifiable, preventable, and serious in their consequences — by the Center for Medicare and Medicaid Services (CMS), as well as numerous authors [5–7].

Falls are not exclusive to older citizens, but elderly patients have an elevated risk for falls [8]. There are many reasons for increased risk of falls with aging such as reduced muscle strength, deterioration in balance control, and use of prescription drugs which can cause dizziness. To address this problem, the first step is identification of those at risk so that specific fall prevention resources, such as fall prevention equipment allocation, physical therapy, or medication reconciliation, can be efficiently allocated.

To identify patients at high risk for falls, diverse groups have developed tools through complex computational methods. Developing these tools is challenging since coded data is not always reliable and outpatient data is often missing [9]. Furthermore, assessing individual risks in any clinical setting remains complex [10]. Given the close relationship between balance, gait, and the occurrence of falls, several groups have studied using machine learning (ML) models to predict falls using sensor data and have obtained promising results [11–13]. However, not all clinical settings are able to provide patients with high-tech devices and sensors, nor can we expect patients to purchase such devices for themselves. Adding to the cost and availability challenges these devices pose, their efficacy is also dependent on patients’ compliance, capacity, and their technological literacy and proficiency [14–17].

For this scoping review, machine learning was defined as a collection of computational methods that automatically learn patterns and relationships from data—in this case, routinely collected clinical records—to build predictive models capable of forecasting outcomes such as the risk of falls in elderly populations.

This study aims to assess the current literature on machine learning algorithms to predict the risk of falls in elders, without using sensor data, and provide guidance for future implementation.

## Methods

A scoping review was conducted following the Preferred Reporting Items for Systematic Reviews and Meta-Analyses (PRISMA) approach [18]. Two searches were carried out: the initial search was implemented on June 22, 2022, and included articles from January 01, 2002, to December 31, 2021; the final search was implemented on February 09, 2025, and it included articles from January 01, 2022, to December 31, 2024. The search query consisted of terms considered pertinent by the authors.

### Searches

The search included peer-reviewed publications in PubMed and Scopus. Title, abstracts, and indexed fields were searched in two searches operated at two different times. The first search was launched on June 22, 2022, using the following string:

- PubMed: “((Elde* OR old OR senil*) AND (fall OR trip) AND (Machine Learning OR"AI"OR algorithm)) AND (("2002/01/01"[Date - Publication] :"2021/12/31"[Date - Publication]))”.
- Scopus: “((elde$ OR old OR senil$) AND (fall OR trip) AND (machine AND learning OR"AI"OR algorithm)) AND PUBYEAR > 2002 AND PUBYEAR < 2022 AND (LIMIT-TO(SRCTYPE,"j") OR EXCLUDE (SRCTYPE,"p")) AND (LIMIT-TO(DOCTYPE,"ar")) AND (LIMIT-TO(LANGUAGE,"English")) AND (LIMIT-TO(SUBJAREA,"COMP") OR LIMIT-TO(SUBJAREA,"MEDI") OR LIMIT-TO (SUBJAREA,"SOCI"))”

The second search was launched on February 09, 2025, using the following string:

- PubMed: ((Elde* OR old OR senil*) AND (fall OR trip) AND (Machine Learning OR"AI"OR algorithm)) AND (("2021/12/31"[Date—Publication]:"2024/12/31"[Date—Publication]))
- Scopus: ((elde$ OR old OR senil$) AND (fall OR trip) AND (machine learning OR"AI"OR algorithm)) AND PUBYEAR > 2021 AND PUBYEAR < 2025 AND (LIMIT-TO(SRCTYPE,"j") OR EXCLUDE(SRCTYPE,"p")) AND LIMIT-TO(DOCTYPE,"ar") AND LIMIT-TO(LANGUAGE,"English") AND (LIMIT-TO(SUBJAREA,"COMP") OR LIMIT-TO(SUBJAREA,"MEDI") OR LIMIT-TO(SUBJAREA,"SOCI"))

#### Study inclusion and exclusion criteria

Studies were included if they focused on predicting fall risk with machine learning in elderly populations and were written in English. For the purposes of this review, “elderly” was defined as individuals aged 65 years or older. A sensitive inclusion approach was adopted: articles were included if the study population included individuals aged 65 or above, even if the overall cohort also included younger participants.

Studies were excluded if they did not focus on the prediction of falls in elders or if they predicted risk by relying on sensor data. Systematic reviews, meta-analyses, opinion papers, case reports, and editorials were excluded.

### Data extraction

Data was extracted by one reviewer (A. C.) and double checked by another (A. S.).

Descriptive variables extracted from each article were as follows: publication year, first author’s affiliation (country and country’s income level), population number, population characteristics (community dwelling, inpatients, age range, main pathology, ethnicity/race), number and name of key variables used in the final ML model, subsets of variables created by authors (if present), and number of models and their results.

Regarding models’ results, “accuracy” (proportion of correctly classified instances or predictions out of the total number of instances evaluated), and area under the receiving curve and receiver operating characteristic (AUC and ROC) were extracted.

## Results

**Fig. 1:**
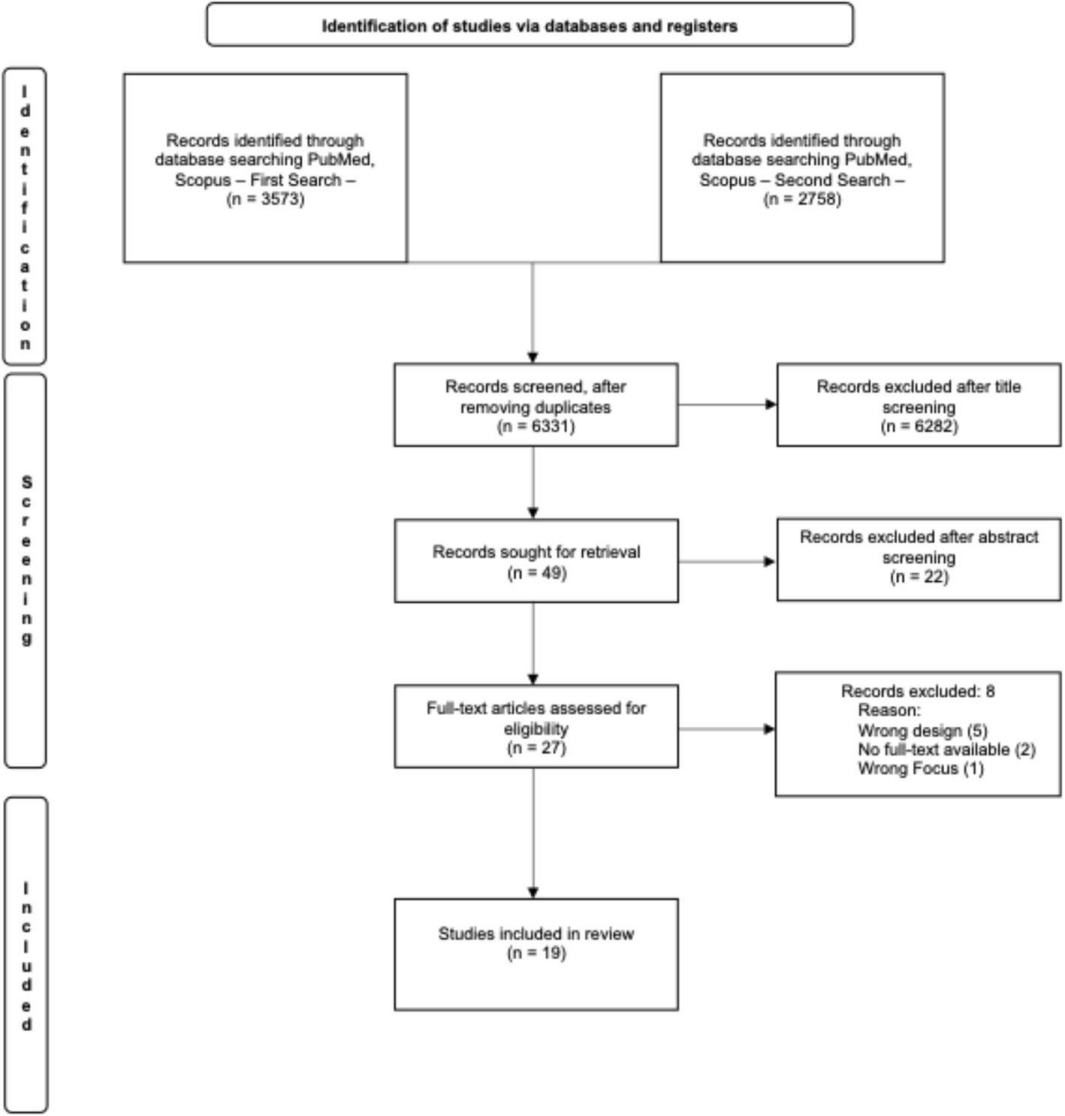
PRISMA flowchart

Figure 1 shows the PRISMA flowchart for the included studies. Table 1 shows the included studies. Out of the 19 included studies [19–37] (Fig. 1), 16 (84%) were published by authors affiliated with high-income countries as defined by the World Bank [38], with Japan and the USA tying first place with 4 studies each.

**Table 1:** Included studies

*Title*	*First author*	*Publication year*	*First author’s affiliation country*	*No. of subjects studied*	*Age range*	*Time horizon for prediction*
*Elderly fall risk prediction based on a physiological profile approach using artificial neural networks*	J. Razmara	2018	Iran	200	> 60	N/A
*A decision model to predict the risk of the first fall onset*	T. Deschamps	2016	France	426	> 65	12 months
*Falling in the elderly: Do statistical models matter for performance criteria of fall prediction? Results from two large population-based studies*	A. Kabeshov	2016	France	3525	> 65	12 months
*Artificial neural network and falls in community dwellers: a new approach to identify the risk of recurrent falling*	A. Kabeshov	2015	France	3289	> 65	12 months
*Simplified decision‐tree algorithm to predict falls for community‐dwelling older adults*	K. Makino	2021	Japan	2520	> 65	48 months
*Serious falls in middle-aged veterans: development and validation of a predictive risk model*	J. A. Womack	2020	USA	275,940	45–65	6 months
*Deep learning prediction of falls among nursing home residents with Alzheimer’s disease*	M. Suzuki	2020	Japan	42	> 80	10 months
*Predicting inpatient falls using natural language processing of nursing records obtained from Japanese electronic medical records: case–control study*	H. Nakatani	2020	Japan	743	> 50	12 months
*Training and interpreting machine learning algorithms to evaluate fall risk after emergency department visits*	B. W. Patterson	2019	USA	9687	> 65	6 months
*Predicting falls in people aged 65 years and older from insurance claims*	M. L. Homer	2017	USA	120,881	> 65	12 months
*A machine learning-based fall risk assessment model for inpatients*	C. H. Liu	2021	China	108,940	40–77	In-hospital stay
*A model for predicting fall risks of hospitalized elderly in Taiwan—a machine learning approach based on both electronic health records and comprehensive geriatric assessment*	W. M. Chu	2022	Taiwan	1101	≥ 65	In-hospital stay
*Exploratory analysis using machine learning of predictive factors for falls in type 2 diabetes*	Y. Suzuki	2022	Japan	226	49–68	12 months
*Factors associated with fall risk of community-dwelling older people: a decision tree analysis*	K. N. K. Fong	2023	Hong Kong	1151	≥ 65	12 months
*Inhospital fall prediction using machine learning algorithms and the Morse fall scale in patients with acute stroke: a nested case–control study*	J. H. Choi	2023	South Korea	1090	56–80	In-hospital stay
*Predicting fall risk in elderly individuals: a comparative analysis of machine learning models using patient characteristics, functional balance tests, and computerized dynamic posturography*	E. Soylemez	2024	Turkey	120	65–79	12 months
*Predicting falls in long-term care facilities: machine learning study*	R. Thapa	2022	USA	2785	≥ 60	3 months
*Predicting falls-related admissions in older adults in Alberta, Canada: a machine-learning falls prevention tool developed using population administrative health data*	V. Sharma	2023	Canada	224,445	≥ 65	12 months
*Using conditional inference forests to examine predictive ability for future falls and syncope in older adults: results from The Irish Longitudinal Study on Ageing*	O. A. Donoghue	2023	Ireland	4706	50–93	48 months

High-income countries had collectively drawn data from a total of 631,696 subjects (min: 42 patients, max: 275,940), while upper-middle-income countries, namely China, Iran, and Turkey, used data from 109,260 subjects (min: 120, max: 108,940). Twelve of 19 studies (63%) [19–22, 24, 26, 27, 29, 30, 33, 34, 37] presented data from non-hospitalized individuals (total: 425,230), and 7 studies [23, 25, 28, 31, 32, 35, 36] presented data from inpatients, totaling 315,726 patients.

In the 19 studies assessed, the population was either enrolled inside the hospital (inpatients) or through the community the subjects lived in (community dwellers). None of the community dwellers (425,230 subjects) were enrolled for a specific pathology. Thus, no disease could be differentiated as a singular confounding factor. The inpatient group consisted of 315,726 subjects, of which 1090 were enrolled after a stroke, 226 were enrolled because of their type 2 diabetes, and 42 patients were enrolled because of their Alzheimer’s disease diagnosis. The remainder was not specified by the authors of the included studies.

A total of 455 variables were included in the best-performing models. We divided these variables into 19 categories, the full extent of which can be found in Table S1. The most common variables were related to miscellaneous conditions, ranging from “handgrip strength score” to “cancer” to “visit in December”; the second most common type of variable was related to cardiovascular measurements/diseases, closely followed by mobility- and equilibrium-related variables.

More than 65 models were described in the aforementioned studies. The highest-performing models and their accuracies and/or AUCs have been summarized in Table 2.

**Table 2:** Models used and their performances

*Authors (year)*	*Model*	*Accuracy*	*AUC*	*Sample size*	*Validation*
*A. Kabeshova (2015) * [21]	NEAT	88%	0.7	3289	Holdout
*T. Deschamps (2016)* [26]	Decision tree	82%	N/A	426	Holdout
*A. Kabeshova (2016)* [20]	ANFIS	87%	0.7	3525	Cross-validation
*M. L. Homer (2017)* [24]	Lasso logistic regression	N/A	0.71	120,881	Holdout
*J. Razmara (2018)* [19]	ANN (psychological and public factors)	91%	N/A	200	Holdout
*B. W. Patterson (2019)* [29]	Random forest	N/A	0.78	9687	Holdout
*J. A. Womack (2020)* [22]	Logistic regression	N/A	0.76	275,940	Holdout
*M. Suzuki (2020)* [28]	CNN “triple factor” (mini-mental state + normalized knee extension strength + FIM locomotion)	65%	N/A	42	Cross-validation
*H. Nakatani (2020)* [23]	MCMC	N/A	0.83	743	Holdout
*K. Makino (2021)* [27]	Decision tree	65%	0.7	2520	Cross-validation
*C. H. Liu (2021) * [25]	Bagging + SVM	71%	0.72	108,940	Cross-validation
*W. M. Chu (2022)* [31]	Random forest	73%	0.69	1101	Cross-validation
*Y. Suzuki (2022)* [32]	Logistic regression	77%	0.75	226	Cross-validation
*R. Thapa (2022)* [30]	eXtreme Gradient Boosting	N/A	0.85	2785	Holdout
*K. N. K. Fong (2023)* [33]	Decision tree	77%	N/A	1151	Cross-validation
*J. H. Choi (2023)* [35]	XGBoost	N/A	0.85	1090	Cross-validation
*V. Sharma (2023)* [36]	CatBoost	N/A	0.7	224,445	Holdout
*O. A. Donoghue (2023)* [34]	Conditional inference forest	67%	0.69	4706	Cross-validation
*E. Soylemez (2024) * [37]	Support vector machine (SVM) and Naïve Bayes	100%	1	120	Holdout

The three most represented model categories were neural network models (14 out of 70 occurrences, 20%), logistic regression variants (13 out of 70 occurrences, 19%), and random forest approaches (11 out of 70 occurrences, 16%). The highest performing model was an SVM that achieved an AUC of 1.0 and 100% accuracy (hold-out validation method).

Some studies, such as the one published by Razmara et al. [19], had summarized variables. These variable summaries, along with three others [24, 28, 29], can be found in Supplemental Material S2. All variables used and the highest performing used in the included studies can be found in Supplemental Material S3.

The best results in terms of accuracy amidst the cross-sectional studies [19–21, 23–25, 30, 33, 35, 36] were described by Razmara et al., who for their best model reported 91.3% accuracy (using NN, with psychological and public factors).

The best results in terms of accuracy and AUC amidst the cohort studies [22, 26–29, 31, 32, 34, 37] were described by T. Deschamps et al., P. W. Patterson et al., and Soylemez et al. who reported respectively 82% accuracy (using decision tree, with an hold-out validation method) and a median AUC of 0.76 across all models (using random forest and variously penalized logistic regressions, with an hold-out validation method) and *AUC* = 1 and 100% accuracy (Naïve Bayes, with an hold-out validation method). They enrolled, respectively, 426, 9687, and 120 patients [26, 29, 37].

On the other hand, the best model’s poorest results were described by Suzuki et al. [28] who obtained an accuracy of 65% using a CNN and just three factors (mini-mental state + normalized knee extension strength + FIM locomotion).

However, we note that these machine learning model results are not directly comparable to each other, as they were fitted on different datasets, with potentially different distributions.

## Discussion

The results highlight significant variations in the prediction of falls among patients, with diverse approaches and model performances across the included studies. Notably, most studies originated from high-income countries, drawing data from a substantial number of subjects. Variable categories ranged from cardiovascular measurements to psychological factors, reflecting the multifaceted nature of fall prediction. While the neural network model incorporating psychological and public factors achieved a remarkable 91.3% accuracy, other models showed varying degrees of success. These findings underscore the complexity of fall prediction and the influence of model selection and variables on outcomes.

Nonetheless, unbiased comparisons are not entirely possible here, since not all algorithms were directly compared over the same datasets, potentially leading to unfair comparisons.

The most studied population was people aged 60 and older, and no study included subjects younger than 40. This pattern was expected as falls are more common in elders, often resulting in serious injuries. Furthermore, given how most falls happen during common day-to-day activities [39], most included studies focused on data collected from community-dwelling adults, and just seven studies analyzed inpatients. This result underlines how prediction based on clinical data might improve community care, potentially lightening the burden on hospitals; however, falls were usually defined as “self-reported” in community-dwelling studies, therefore limiting the analyzed studies findings’ reliability.

Analyzed studies were most commonly published by high-income countries; therefore, their conclusions should be valued in their own demographics, and since there were no multicentric validations or piloted attempts at clinical implementation, we cannot generalize their results to the global population.

### Complex vs. simple algorithms

In analyzing the performance of various machine learning models, it becomes evident that model complexity and the availability of training data can influence predictive accuracy. Simpler models, such as decision trees and logistic regression, have shown robust performance, particularly when trained on limited datasets. For instance, Deschamps et al. [26] employed a decision tree algorithm achieving an accuracy of 82% with a sample size of 426, while Womack et al. [22] utilized logistic regression to attain an AUC of 0.76 on a substantially larger sample of 275,940 individuals. Conversely, more complex models like neural networks seem to require substantial amounts of data to perform effectively. Suzuki et al. [28] implemented a CNN using only 42 samples (and bootstrapping them), resulting in a lower accuracy of 65%. This underscores the necessity for large training data when deploying neural networks, as insufficient data can lead to suboptimal performance. Notably, when complex models are trained on extensive datasets, they can achieve superior predictive capabilities. For example, Sharma et al. [36] applied a CatBoost algorithm to a validation dataset of 203,584 samples, resulting in an AUC of 0.70. Similarly, Thapa et al. [30] utilized eXtreme Gradient Boosting on 2785 samples, achieving an AUC of 0.85.

These observations align with existing literature, which empirically suggests that while complex models such as neural networks have the potential for higher predictive power, they necessitate larger datasets to realize this potential [40–42]. Selecting an appropriate machine learning model for fall prediction in the elderly should involve an assessment of model complexity relative to the available training data. Simpler models may offer sufficient performance with limited data and are easier to implement, whereas complex models may provide enhanced accuracy when supported by large, high-quality datasets.

### Common and important variables

The most common homogeneous variables were mainly related to cardiovascular status and mobility assessed without sensors (see Supplemental S1). A selection of other variables, classified as “miscellaneous,” often correlated with the risk of falls and ranged from “arrived by self or with family” to “day of the week for the visit” and “drugs for peptic ulcer.”

The variety of predictors highlights the need for broad inclusions to allow for the discovery of important but unexpected variables.

Table S3 summarizes the most important variables per each study. The five most important variable categories were respectively mobility/equilibrium, miscellaneous, cardiovascular, mental health, and demographic data. We believe this last set of variables might be worthy of future research, as it underlines how social determinants of health are associated with falls.

### Fairness

Fairness and minimizing bias—understood here in both its statistical and social dimensions—are increasingly recognized as critical to precision medicine. Here, we refer to statistical bias as to systematic errors in model performance, while social bias denotes the unfair or inequitable treatment that may result when such errors disproportionately harm underrepresented groups [43]. It is an increasing movement to dismantle the hard coding of sensitive attributes in AI-/ML-derived tools, with much attention focused on race and ethnicity. This transition necessitates a more deliberate and thoughtful approach to model design, underpinned by a deeper understanding of how various social determinants of health contribute to data-driven outcomes in medical decision-making [44]. This was consistent with our findings since one of the best achieving algorithm, achieved by Razmara et al. [19], used “public factors,” which included age, gender, education level, and employment among others, but not race; recognizing and addressing bias in healthcare algorithms necessitate a holistic approach that encompasses not only racial bias but also factors related to other social determinants of health. We argue that using more social determinants of health variables could considerably increase the models’ fairness and their outputs.

### Common gaps

This study found the analysis of algorithms’ performance was inconsistent across studies. The extraction of accuracy, whenever possible, may not be the best measure of performance evaluation. Accuracy is a single measure and can be misleading when presented without event distribution information, potentially leading to misinterpretations. For example, if the entire population has an event, then a faulty algorithm only capable of one positive output will always have 100% accuracy. This was not the case with the included studies, but more transparent and homogeneous reporting can prevent low-quality science. Additionally, a probability threshold has to be determined to classify a fall from a non-fall, which is not necessarily trivial. Metrics like AUROC [45] or the area under the precision-recall curve (AUPRC) are threshold agnostic and might be the better choice to represent discrimination performance. Adopting transparent reporting practices and considering threshold-agnostic metrics like AUROC and AUPRC are essential steps towards ensuring robust and meaningful evaluations of algorithm performance in healthcare studies.

### Limitations

This study comes with a few limitations. It is possible that our search strategy may not have included certain studies, especially those published in computer science journals not indexed in the searched databases. Furthermore, other studies may have been missed due to the keywords used in our search strategy, the incompleteness of which is often embedded in reviews. Additionally, a notable limitation of this review is the heterogeneity in the age criteria used across the included studies. While most studies defined the elderly population as individuals aged 65 years or older, some studies enrolled participants beginning at younger ages (e.g., 40, 45, or 50 years). This variation may introduce variability in the fall risk profiles and could potentially affect the generalizability of our findings to a uniformly defined elderly population.

## Conclusions

This scoping review found that machine learning models using commonly recorded data can predict falls with high accuracy, as reported by the individual studies. The results highlight the importance of understanding the diverse factors influencing the model’s performance. The predominance of high-income countries in both research and participant representation underscores the need for a broader global perspective in this field. The variation in study populations, from community dwellers to inpatients with specific diagnoses, highlights the complexity of fall prediction and the necessity for tailored approaches. Moreover, the success of neural network models incorporating psychological and public factors demonstrates the potential for multifaceted models to enhance accuracy.

These findings offer a valuable overview of fall prediction models’ performance and variability, setting the stage for further advancements in this crucial area of healthcare research.

## Supplementary Information

Supplementary Material 1. Supplemental Material S1: Variables included in the best performing models.

Supplementary Material 2. Supplemental Material S2: Variable summaries

Supplementary Material 3. Supplemental Material S3: All variables used and the highest performing used in the included studies

## Data Availability

No datasets were generated or analysed during the current study.

## Abbreviations

AI: Artificial intelligence
AUC: Area under the receiving curve
AUPRC: Area under the precision-recall curve
AUROC: Area under the receiver operating characteristic curve
CI: Confidence interval
CMS: Center for Medicare and Medicaid Services
ML: Machine learning
MLP: Multilayer perceptron
MCMC: Markov Chain Monte Carlo
NN: Neural network
PRISMA: Preferred Reporting Items for Systematic reviews and Meta-Analysis
ROC: Receiver operating characteristic
SES: Socioeconomic status
SVM: Support vector machine
USA: United States of America
