# Supplementary material for: A scoping review of machine learning models to predict risk of falls in elders, without using sensor data
Source: Diagn Progn Res. 2025 May 6;9:11. doi: 10.1186/s41512-025-00190-y (PMC12054167; doi:10.1186/s41512-025-00190-y)
Supplement: Supplementary file 2 — Supplementary Material 2. Supplemental Material S2: Variable summaries [file 41512_2025_190_MOESM2_ESM.docx]

| **Title** | **Model Used** | **Summarization** |
| --- | --- | --- |
| Elderly fall risk prediction based on a physiological profile approach using artificial neural networks | ANN, modeled with data from **Public Factors**, **Psychological Factors**, or both | **Psychological factors**: Cleaning the house, Create a simple meal, Go to the store, Go up or down the stairs, Getting hands above head, Walking on slippery surfaces, Walking in a crowded places, Go up or down the slope, Dressing or undressing, Bathing or showering, Sit down or get up from the chair, Walking, Answering phone, Go to visit friends or family, Walking on an uneven surface, Going out to gathering.  **Public factors:** Age, Sex, Education, Employment, Living conditions, Life independency, Fear of falling, Use of drug, Needs to help for movement, Financial condition, Chronic diseases, Cardiovascular diseases, Muscular diseases, Respiratory diseases, Metabolic diseases, Neurological diseases. |
| Deep learning prediction of falls among nursing home residents with Alzheimer's disease | CNN, modeled by **Single Factor, Double Factor, Triple Factor**. | **Single Factor**: FIM (Functional Independence Measure); KES (normalized knee extension strength); MMSE (Mini-Mental State Examination).  **Double Factor**: AGE-KES, AGE-MMSE, AGE-FIM, KES-MMSE, KES-FIM, MMSE-FIM.  **Triple Factor:** AGE-KES-FIM, AGE-KES-MMSE, AGE-MMSE-FIM, KES-MMSE-FIM |
| Predicting Falls in People Aged 65 Years and Older from Insurance Claims | Lasso Logistic Regression, modeled by **Demographics**, **Drugs** and **Diagnoses**. | **Demographics**: Age in years at the beginning of the observation period, gender.  **Drugs**:  Antidementia drugs Dopaminergic agents  Antipsychotics  Antidepressants  Laxatives  High-ceiling diuretics  Antiepileptics  Antithrombotic agents  Opioids  Vasodilators used in cardiac diseases  Drugs for peptic ulcer and GORD  Arteriolar smooth muscle, agents acting on  Other analgesics and antipyretics  Low-ceiling diuretics, excl. thiazides  Hypnotics and sedatives  Antiadrenergic agents, centrally acting  Potassium-sparing agents  Muscle relaxants, centrally acting agents  Peripheral vasodilators  Antiadrenergic agents, peripherally acting  Anxiolytics  Low-ceiling diuretics, thiazides  Psychostimulants, agents used for ADHD and nootropics  Antispasmodics in combination with psycholeptics  Antiadrenergic agents, ganglion-blocking  Other antihypertensives  Antihypertensives and diuretics in combination  Combinations of antihypertensives in Atc-Gr. CO_2_  Diuretics and potassium-sparing agents in combination  Other diuretics  Muscle relaxants, peripherally acting agents  Psycholeptics and psychoanaleptics in combination  Antivertigo preparations Muscle relaxants, directly acting agents  **Diagnosis**:  Delirium, dementia, and amnestic and other cognitive disorders  Schizophrenia and other psychotic disorders  Central nervous system infection  Hereditary and degenerative nervous system conditions  Mood disorders  Other nervous system disorders  Epilepsy; convulsions  Syncope  Anxiety disorders  Sleep disorders  Conduction disorders  Cerebrovascular disease  Malaise and fatigue  Cardiac dysrhythmias  Osteoarthritis  Urinary tract infections  Spondylosis and allied disorders  Diabetes with neurological manifestations  Conditions associated with dizziness or vertigo  Eye disorders  Cancer of brain and nervous system  Osteoporosis  Diabetes with ophthalmic manifestations  Secondary malignancy of brain/spine  Motor skill disorders  Hypotension |
| Training and Interpreting Machine Learning Algorithms to Evaluate Fall Risk After Emergency Department Visits | Of the 725 variables used for every model, only the top features were listed. | **Top ADABoost:**  age  number of hospitalizations in prior 6 months  arrived by self or with family/friends  count of Elixhauser comorbidities = 0  no history of other neurologic disorder  no history of pulmonary circulatory disease  index visit not for fall  Hendrich II get up and go score = 0  elevated red blood cell distribution width (sd)  count of primary care visits in past 6 months  count of specialty care visits in past 6 months  length of ED stay  average diastolic blood pressure in ED  first diastolic blood pressure in ED  last diastolic blood pressure in ED  maximum diastolic blood pressure in ED  minimum diastolic blood pressure in ED  average pulse oximetry value in ED  first pulse oximetry value in ED  last pulse oximetry value in ED  **TOP Ridge Logistic Regression**  history of pulmonary circulatory disease  arrived via EMS  history of neurologic disorder  index visit for fall  no history of renal failure  elevated platelet count  number of hospitalizations in prior 6 months  ED arrival 19:00-20:00  no history of lymphoma  Hendrich II get up and go score = 3  visit in December  diagnosis of urinary retention  chief complaint of “hip pain”  low CO2 level  normal thyroid stimulating hormone level  elevated magnesium level  elevated red blood cell distribution width (sd)  visit on Wednesday  chief complaint of “leg pain”  count of Elixhauser comorbidities = 1  **TOP Lasso Logistic regression**  no history of lymphoma  history of pulmonary circulatory disease  elevated platelet count  history of neurologic disorder  no history of renal failure  index visit for fall  Prescribed medication in therapeutic class “cardiovascular”  no prescribed medication in therapeutic class “genitourinary”  ESI acuity = 5  elevated magnesium level  ED arrival 19:00-20:00  Low CO2 level  Normal thyroid stimulating hormone level  chief complaint of “hip pain”  elevated ketones in urinalysis  chief complaint of “leg pain”  count of Elixhauser comorbidities = 1  administered medication in therapeutic class “gastrointestinal agents”  female gender  **Top logistic regression:**  normal pH  no prescribed medication of class angiotensin receptor blocker  no history of peptic ulcer disease  no prescribed antiviral medication  missing hematocrit  no history of blood loss anemia  missing hemoglobin A1C value  missing renal epithelial cells urinalysis value  no prescribed beta blocker  elevated serum bicarbonate level  no administered medication of type “study drug”  no prescribed anti-dementia drug  ESI acuity = 5  missing serum creatinine level  missing estimated glomerular filtration rate  missing mean corpuscular volume  missing white blood cell count  normal basophil count  no prescribed calcium channel blocker  low red blood cell distribution width (sd)  **TOP random forest:**  age  number of PCP visits in baseline  arrived by self or with family/friends  arrived via EMS  average systolic blood pressure in ED  last measured systolic blood pressure in ED  first measured diastolic blood pressure in ED  minimum systolic blood pressure in ED  first systolic blood pressure in ED  average diastolic blood pressure in ED  average heart rate in ED  maximum systolic blood pressure in ED  average pulse oximetry value in ED  first heart rate in ED  count of hospitalizations in prior 6 months  maximum diastolic blood pressure in ED  maximum heart rate in ED  last diastolic blood pressure in ED  first heart rate in ED  minimum heart rate in ED |
