# Supplementary material for: A scoping review of machine learning models to predict risk of falls in elders, without using sensor data
Source: Diagn Progn Res. 2025 May 6;9:11. doi: 10.1186/s41512-025-00190-y (PMC12054167; doi:10.1186/s41512-025-00190-y)
Supplement: Supplementary file 3 — Supplementary Material 3. Supplemental Material S3: All variables used and the highest performing used in the included studies [file 41512_2025_190_MOESM3_ESM.docx]

| Article and Author | Best Performing Model Used | Most Important Predictors |
| --- | --- | --- |
| Elderly fall risk prediction based on a physiological profile approach using artificial neural networks   - J Razmara et al. | NN | use of drug; fear of falling; metabolic diseases; cardiovascular diseases; employment; go up or down the slope; walking on slippery surfaces; walking in a crowded place; bathing or showering; walking on an uneven surface |
| A decision model to predict the risk of the first fall onset   - T Deschamps et al. | Decision Tree | Mini-Nutritional Assessment score; lean mass; BMI=21.85; BMI=23.25; ankle paresthesia; EC surface=292.5 mm2; EC AP length 845.3mm; limited knee range motion; EC surface= 381.5mm2; EO ML length= 311.2mm; foot pathology; age=73.5; EO surface= 93.17mm2; visual acuity; presbycusis; EC Surface= 269mm2. |
| Falling in the elderly: Do statistical models matter for performance criteria of fall prediction? Results from two large population-based studies   - A Kabeshova et al. | ANFIS | height; weight; BMI; Number of prescriptions; vision score; TUG |
| Artificial neural network and falls in community-dwellers: a new approach to identify the risk of recurrent falling?   - A Kabeshova et al. | NEAT | BMI < 21 kg/m2; fear of falling; Female gender; Depression; Cognitive disorders; Use of calcium; Use of walking aid; Use of psychoactive drugs; TUG; Age ≥ 75; Use of vitamin D supplements; vision; Lower-limb proprioception; Handgrip strength score; Use of diphosphonate; Number of drugs daily taken |
| Simplified decision‐tree algorithm to predict falls for community‐dwelling older adults   - K Makino | Decision Tree | age (grouped); fall history; fear of falling; polypharmacy; knee osteoarthritis; gait speed |
| Serious Falls in Middle-Aged Veterans: Development and Validation of a Predictive Risk Model   - JA Womack | Logistic Regression | Fall within the past 12 months; Count of chronic medications; Count of physical comorbidities; Non-White race; Female; Diagnosis of alcohol use/abuse; Pain; Count of mental health comorbidities; Hazardous alcohol use; Fall within the past 12 months * Count of physical comorbidities; Opioid prescription; BMI; Benzodiazepines; VACS Index Score 2.0; Female* Count of chronic medications; Anticonvulsants; Selective; Serotonin; Reuptake Inhibitors; Fall within the past 12 months*diagnosis of alcohol use/abuse; Fall within the past 12 months* pain; Muscle Relaxants Female*Fall within the past 12 months; Fall within the past 12 months* pain; Fall within the past 12 months*diagnosis of alcohol use/abuse; Fall within the past 12 months* Count of physical comorbidities |
| Deep learning prediction of falls among nursing home residents with Alzheimer's disease   - M Suzuki | NN | Age; Functional Independence Measure; Normalized knee extension strength; Mini-Mental State Examination. |
| Predicting inpatient falls using natural language processing of nursing records obtained from Japanese electronic medical records: Case-control study   - H Nakatani | MCMC | 378 morphemes derived from NLP |
| Training and Interpreting Machine Learning Algorithms to Evaluate Fall Risk After Emergency Department Visits   - BW Patterson | ADABoost and Ridge Logistic Regression | See Supplemental Material S-2. |
| Predicting Falls in People Aged 65 Years and Older from Insurance Claims   - ML Homer | LASSO Logistic Regression | See Supplemental Material S-2. Combination of sets. |
| A Machine Learning-Based Fall Risk Assessment Model for Inpatients   - CH Liu | Bagging Support Vector Machine | fall history; walking ability; depression; self-care: toileting; fall scale score; hypnotic drugs; stroke; high risk group of fall; consciousness; lower limbs muscle strength |
| A model for predicting fall risks of hospitalized elderly in Taiwan-A machine learning approach based on both electronic health records and comprehensive geriatric assessment   - Chu WM | Random Forest | Male; Age; Weight; Height; Diastolic pressure; Systolic pressure; Heart rate; Respiratory rate; ADL; MNA; Brade score; IADL; VAS; CHS; Polypharmacy; Psychiatric medication; Visual impairment; Hearing impairment; Difficulty in communication; Sleep disturbance; Urinary incontinence |
| Exploratory analysis using machine learning of predictive factors for falls in type 2 diabetes   - Suzuki Y | Logistic Regression | Knee extension strength; Fasting C‑peptide (F‑CPR) level; Dorsiflexion strength |
| Factors associated with fall risk of community-dwelling older people: A decision tree analysis   - Fong KNK | Decision Tree | Demographics & social factors: age; gender; education; living environment; living arrangement; dependency for household duties; social support.  Clinical/health measures: presence of chronic diseases; various comorbidities (e.g., osteoporosis; depression; previous fractures; etc.); number of drugs taken; blood pressure.  Functional assessments: Timed Up and Go (TUG) test; Functional Reach (FR) test; body mass index (BMI); visual acuity; among others. |
| In-hospital fall prediction using machine learning algorithms and the Morse fall scale in patients with acute stroke: a nested case-control study   - Choi JH | Extreme-Gradient Boosting | Ward type; Ambulation method; Diabetes; Arrhythmia; Degenerative spinal disease; Cerebral neurodegenerative disease; Medication use; Dyslipidemia; Mental status. |
| Predicting Fall Risk in Elderly Individuals: A Comparative Analysis of Machine Learning Models Using Patient Characteristics, Functional Balance Tests, and Computerized Dynamic Posturography   - Soylemez E | Naive Bayes | Height; Weight; Body mass index (BMI); Imbalance symptoms; Imbalance severity; Duration of imbalance; Hypertension; Diabetes mellitus; Right ear pure tone audiometry threshold; Left ear pure tone audiometry threshold; Hearing loss categorization; Asymmetric hearing loss; Traditional Romberg test; Foam pad Romberg test; Tandem Romberg test; One‐leg standing test (eyes open); One‐leg standing test (eyes closed); Timed Up and Go (TUG) test; Functional Reach Test (FRT); Tinetti Balance and Gait Test (TBGT); SOT1; SOT2; SOT3; SOT4; SOT5; SOT6. |
| Predicting Falls in Long-term Care Facilities: Machine Learning Study   - Thapa R | Extreme-Gradient Boosting | Age; Sex; Time since last fall; Hypertension; Chronic heart failure; Stroke/Cerebrovascular disease; Benzodiazepines; ACE inhibitors; Antiepileptics; Total number of active medications; Height; Weight; History of lower extremity fracture/dislocation; Diastolic blood pressure; Systolic blood pressure; Heart rate; Respiratory rate; Temperature; Minimum; Maximum; Mean; Standard deviation; Last value; Number of measurements. |
| Predicting falls-related admissions in older adults in Alberta, Canada: a machine-learning falls prevention tool developed using population administrative health data   - Sharma V | CatBoost | Age; Sex; Geographic location; Mean income; Drug dispenses in the previous year using ATC codes level 3 or 4; Opioid use; Benzodiazepine use; Polypharmacy; Prior medical history; Frailty markers; Cardiac disorders; Pulmonary disorders; Diabetes; Hypertension; Neurologic disorders; History of injury; Cancer; Peptic ulcer disease; Renal disease; Anemia; Electrolyte disorders; Lipidemia; Rheumatic disorders; Psychiatric disorders; History of falls; Dementia; Liver disorder; HIV; Urinary tract disorders; Number of unique health providers; Prior hospital admissions; Emergency department visits; Total days in hospital; Blood urea nitrogen; Mean corpuscular volume; Hemoglobin; Potassium; Creatinine; eGFR; Sodium; Hematocrit; ALT; Ferritin; Albumin; Hemoglobin A1C; NT-proBNP. |
| Using Conditional Inference Forests to Examine Predictive Ability for Future Falls and Syncope in OlderAdults: Results from The Irish Longitudinal Study on Ageing   - Donoghue OA | Conditional Inference Forest | Unsteadiness; Falls History; Grip Strength; CAGE score; Medications; Chronic Pain; TUG; CGS; CES-D; Fear of Falling; UGS; SBP drop (120s); Incontinence. |
